# Optimizing the East African Community’s Medicines Regulatory Harmonization initiative in 2020–2022: A Roadmap for the Future

**DOI:** 10.1371/journal.pmed.1003129

**Published:** 2020-08-12

**Authors:** Mawien Arik, Emmanuel Bamenyekanye, Adam Fimbo, Joseph Kabatende, Agnes Sitta Kijo, Burhani Simai, Fred Siyoi, Samvel Azatyan, Aggrey Ambali, Emer Cooke, Jane H. Mashingia, John Patrick Mwesigye, Margareth Ndomondo-Sigonda, Hiiti Sillo, Stanley Sonoiya, Paul Tanui, Mike Ward, Thomas Delano

**Affiliations:** 1 Drug and Food Control Authority, Juba, South Sudan; 2 Directorate of Pharmacy, Medicines, and Laboratories, Bujumbura, Burundi; 3 Tanzania Medicines and Medical Devices Authority, Dar Es Salaam, Tanzania; 4 Rwanda Food and Drugs Authority, Kigali, Rwanda; 5 Zanzibar Food and Drug Agency, Zanzibar City, Zanzibar; 6 Pharmacy and Poisons Board, Nairobi, Kenya; 7 World Health Organization, Geneva, Switzerland; 8 African Union Development Agency–New Partnership for Africa’s Development, Midrand, South Africa; 9 East African Community Secretariat, Arusha, Tanzania; 10 Boston Consulting Group, Paris, France

## Abstract

Margareth Ndomondo-Sigonda outlines future challenges for the East African Medicines Regulatory Harmonization initiative.

**Summary Points**

- The East African Community (EAC)’s Medicines Regulatory Harmonization (MRH) initiative was created to improve access to quality, safe medicines in the region by simplifying the regulatory process while maintaining a high level of rigor. Building on lessons learned since its launch in 2012, the EAC MRH initiative has created a Roadmap for the Future.
- The capacity to monitor and reports adverse drug reactions is key for patients’ safety. Therefore, going forward, drug safety and quality surveillance will be a priority for the EAC MRH initiative.
- In the future, other key success factors for the initiative will include establishing a cadre of regional technical officers (RTOs), a Cooperation Framework Agreement between the national medicines regulatory authorities (NMRAs) of EAC Partner States, and a sustainable funding mechanism for regional assessment.
- Widening the scope of medical products considered (and focusing on those not eligible for the World Health Organization [WHO]’s Prequalification Programme) will also add value to the EAC MRH initiative.
- Implementing the EAC MRH initiative’s agreed upon roadmap will lead to a stronger, more efficient, and more accountable region-wide regulatory system, thus improving access to quality, safe medicines for EAC residents.

## Introduction

The combination of artesunate with amodiaquine is widely used to treat malaria in sub-Saharan Africa. However, in 2012, a researcher noticed something concerning: 43 reports in adults and 6 reports in children of acute extrapyramidal reactions associated with the use of this combination [1]. These reports, collected by the World Health Organization (WHO)’s global Individual Case Safety Report VigiBase database, hosted and managed by Sweden’s Uppsala Monitoring Centre, along with data gathered by the manufacturer, resulted in a new labeling and package insert for the medicine combination that warned of the potential for acute extrapyramidal reactions [2]. (Drug-induced extrapyramidal side effects are movement disorders with acute and long-term symptoms, including dystonia, akathisia, parkinsonism, bradykinesia, tremor, and tardive dyskinesia.) Without the help of the eight sub-Saharan African countries that submitted reports to VigiBase, it is unclear whether the association between the medicine combination and this adverse event would have been detected. Because of the countries’ vigilance, healthcare providers can now be alert for these reactions and treat them promptly. Unfortunately, due to a number of factors, including inadequate legal framework and infrastructure, limited financial resources, and the number and competency of staff available to support pharmacovigilance systems, many African countries struggle to gather and report information about suspected adverse events, which is key for monitoring the safety of medicines on the market. Today, fewer than 10% of reports to VigiBase come from low- and middle-income countries, where roughly 84% of the world’s population resides [3,4]. Building a more robust framework for post-marketing surveillance of the safety and quality of medicines is a pressing need in African countries, one that will only become more urgent as a growing number of medicines used primarily by Africans emerges, thanks to the product development partnerships and programs that are currently targeting diseases endemic in low- and middle-income countries.

To ensure that its residents have access to safe, quality medicines, the East African Community (EAC) is expanding its Medicines Regulatory Harmonization (MRH) initiative to include a product safety and quality surveillance workstream, which will include reporting suspected adverse reactions to medicine manufacturers and the WHO. Indeed, this is just one of many areas in which the initiative plans to grow in the coming years. In this article, one of five in a Special Collection about the EAC MRH initiative, we will describe the Roadmap for the Future (“The Roadmap”) that the EAC MRH initiative has created for growing into a mature, stable program capable of meeting more of the region’s regulatory needs by 2022 [5]. We believe that sharing the EAC’s vision for the next phase of the MRH initiative and its plans for achieving that vision may be helpful for other regions embarking on similar initiatives to harmonize regulatory standards, optimize regulatory processes, and increase regional collaboration.

## Creating the Roadmap for the Future, 2020–2022

In 2017, as the end of the 5-year pilot phase of the EAC MRH initiative approached, the program established a task force that worked with the Boston Consulting Group to determine the initiative’s goals for the next 5 years and how those goals could be achieved. In particular, its aim was to identify steps that could be taken (1) over the short term, to improve existing processes and expand into new regulatory areas and activities; (2) in the period between 2020 and 2022, to develop a well-coordinated and well-functioning regional assessment and inspection process, upon which national registration decisions will rely; and (3) over a longer period, to create a sustainable, semiautonomous agency that will provide regulatory guidance and coordination for the entire region by 2022. The task force created a roadmap detailing how the initiative could achieve each of these objectives and presented its plans to all EAC Partner States, as well as key initiative funding and technical partners, such as the African Union Development Agency–New Partnership for Africa’s Development (AUDA-NEPAD), WHO, the Swiss Agency for Therapeutic Products (Swissmedic), and the Bill & Melinda Gates Foundation. After the task force integrated the stakeholder feedback it received, the EAC’s Council of Health Ministers approved the Roadmap in 2018. Since then, the initiative has been carrying out the actions prescribed in the document.

## Major features of the Roadmap for the Future

### Improving existing regulatory activities

In the EAC MRH initiative’s joint process for assessing marketing applications, staff from two national medicines regulatory authorities (NMRAs) are assigned to perform a thorough initial assessment of the product application. They then share their findings for wider discussion by all the EAC’s NMRAs, which in turn decide whether to recommend approval of the product. Ultimately, each NMRA makes its own decision regarding whether to register the product nationally. However, the rigorous product review provided by the joint assessment is intended to substantially reduce the amount of time needed for NMRAs to make their final decisions. By participating in the joint assessment process, manufacturers should be able to more efficiently receive marketing authorization in multiple EAC Partner States. Unfortunately, feedback from users of the initiative’s pilot program for joint product assessments has revealed some challenges, including a lack of easily accessible public information about the process, poor communication from regulators during the joint assessment process, and a significant lag time between a joint assessment recommendation and national registration decisions [5,6]. Similar problems have been reported for the initiative’s good manufacturing practice (GMP) joint inspection program.

**Fig 1:**
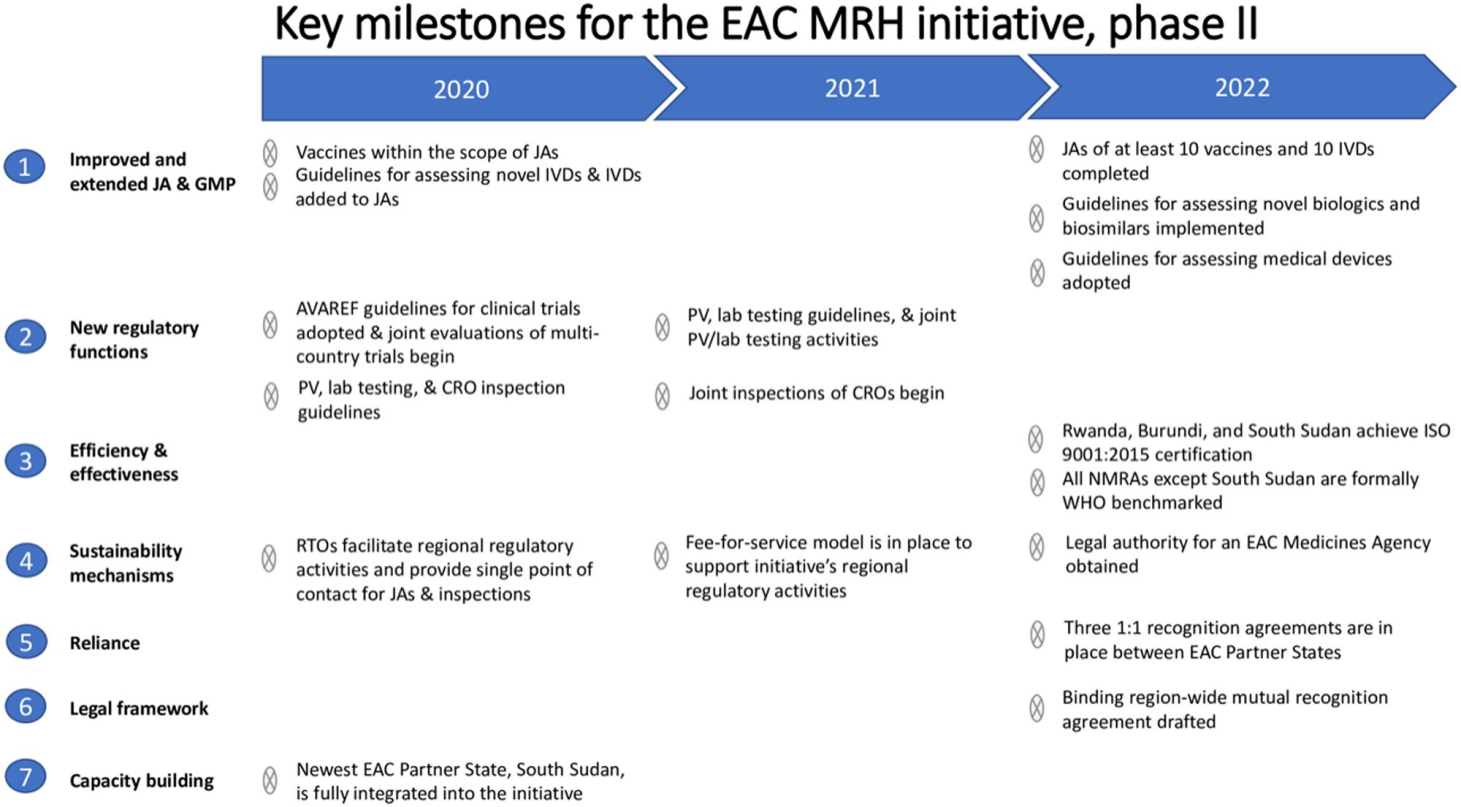
The Roadmap for the Future of the EAC’s MRH initiative, 2020–2022. CRO, contract research organization; EAC, East African Community; GMP, good manufacturing practice; ISO, International Organization for Standardization; IVD, in vitro diagnostic; JA, joint assessment; MRH, Medicines Regulatory Harmonization; NMRA, national medicines regulatory authority; PV, pharmacovigilance; RTO, regional technical officer; WHO, World Health Organization.

To address these problems, the Roadmap (Fig 1) calls for a cadre of regional technical officers (RTOs) who are focused on the day-to-day management of joint activities. In the pilot phase of the EAC MRH initiative, the EAC Secretariat hired one officer for each NMRA to conduct program activities. However, these officers focused primarily on capacity-building activities; often, they and other NMRA staff did not have the time to plan and conduct joint activities efficiently. The RTOs—who will be selected and paid by their home NMRAs—will work alongside the existing program officers and will concentrate solely on facilitating regional regulatory activities (i.e., receiving and screening joint applications, planning joint activity sessions in collaboration with the EAC Secretariat, and sharing application dossiers and joint activity findings with other NMRAs). The RTOs will meet face to face at least once a quarter and will use their expertise in joint activities to recommend programmatic changes to the initiative’s steering committee.

To ensure that all the program’s priorities are being championed, each NMRA now has one RTO, each of whom specializes in a different area. Burundi’s RTO supports clinical trial oversight; Kenya’s, pharmacovigilance; Rwanda’s, information management systems (IMSs); South Sudan’s, policy, legal, and regulatory reforms; Tanzania’s, joint product assessments; Uganda’s, joint GMP inspections; and Zanzibar’s, quality management systems. In April 2019, the EAC Secretariat organized the RTOs’ first meeting, where the officers reviewed the initiative’s progress; developed advocacy and communication materials to increase the visibility of the initiative [7]; and created tools that the NMRAs can use to track joint activity timelines, to increase transparency. The RTOs now provide the single point of contact for joint activities. For example, for a joint product assessment, Tanzania’s RTO will now distribute dossiers to each EAC NMRA, so the applicant does not have to do it themselves. The RTO will also screen the dossier to ensure that all major issues are raised before the joint assessment takes place and work with the EAC Secretariat to plan joint assessments at a time convenient for all parties involved, including technical partners such as Swissmedic or WHO. Finally, once a product has been recommended through the joint assessment process, the RTO will follow up with NMRAs to ensure that national registrations proceed in a timely manner.

The Roadmap also calls for establishing a Cooperation Framework Agreement among the EAC’s Partner States, so that a joint assessment or inspection decision will be honored throughout the region. In May of 2018, the EAC’s Council of Health Ministers approved a Cooperation Framework Agreement for the NMRAs of EAC Partner States [8]. In this document, EAC Partner States agreed to make regulatory decisions in a timely manner based on the outcomes of the initiative’s joint activities; however, final regulatory decisions will remain with individual NMRAs. As an intermediary step toward a binding mutual recognition agreement between all Partner States, the initiative will pursue unilateral recognition agreements, like the one in which Zanzibar’s NMRA agrees to recognize the regulatory decisions of Tanzania’s NMRA, as well as bilateral agreements. By 2022, the program aims to have at least three one-to-one recognition agreements in place, as well as a draft of a binding region-wide mutual recognition agreement.

**Fig 2:**
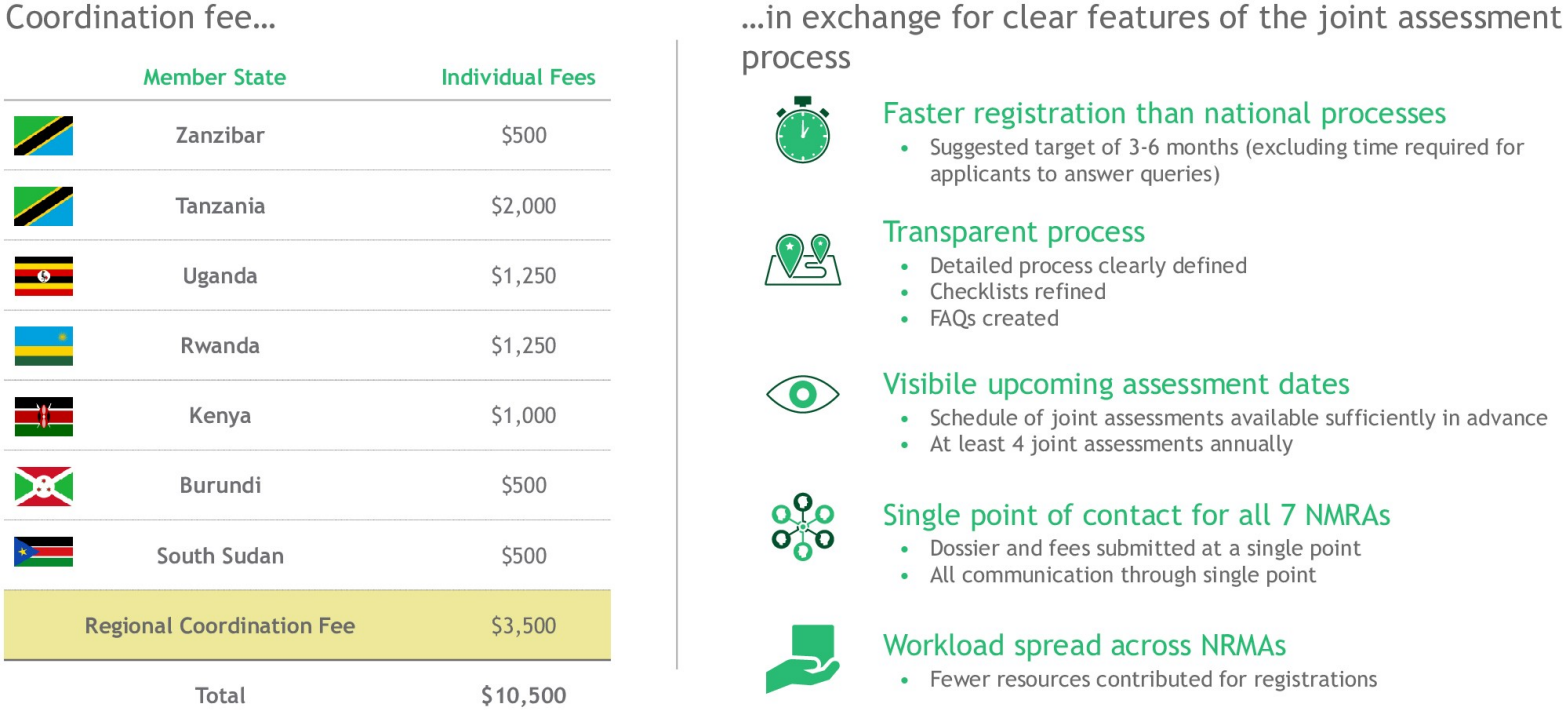
A model for how the EAC’s MRH initiative could finance regional regulatory activities. In this example, an applicant seeking to market their product in the EAC would pay a single fee to a regional coordination desk, which would then divide the money between each Partner State, setting aside a coordination fee for shepherding a single application through the regional product assessment process. A similar model could be used for GMP inspections. These fees are only illustrative and the models are not yet in use, but they are expected to be in place by 2022. EAC, East African Community; FAQs, frequently asked questions; GMP, good manufacturing practice; MRH, Medicines Regulatory Harmonization.

Ultimately, it is envisioned that a coordination fee will be introduced to support regional assessment and inspection processes. Applicants seeking marketing authorization in the EAC could submit a single application and pay a single fee to register their products in every Partner State (Fig 2). The proposed funding scheme would be similar to the one used for the EAC Single Tourist Visa, whereby visitors to Kenya, Rwanda, and Uganda pay a single application fee of $100 at their point of entry. This master fee includes a $10 administration fee for the country that collects the funds and $30 for each of the three countries involved. The simplicity and efficiency of this arrangement would be extremely appealing to pharmaceutical companies.

One of the Roadmap’s long-term goals is to establish a semiautonomous EAC Medicines Agency that is devoted to conducting joint activities. With dedicated staff including an executive director, a joint activity coordinator, a working group coordinator, and an accountant, an EAC Medicines Agency could raise the visibility of the initiative across the region and ensure that the program continues to grow and improve. It would assume responsibility for coordinating joint activities from the EAC Secretariat, which would increase efficiency and convenience for all parties involved. It could also continue to promote a legal framework for reliance on and recognition of joint assessment recommendations, as well as other regulatory work products and decisions, by EAC Partner States. This could allow a user to submit a single application for marketing authorization or GMP certification throughout the EAC. By 2022, the initiative aims to have obtained legal authority for the agency through adoption of a protocol by the EAC Council of Ministers.

### Expanding to new Partner States, medical products, and regulatory activities

In 2016, the EAC welcomed its newest Partner State: South Sudan. The Roadmap includes plans to fully integrate this new member into the EAC MRH initiative in 2020. South Sudan’s NMRA has been working with Tanzania’s NMRA to quickly build an effective regulatory system, even amidst challenging domestic realities. The Roadmap contains a comprehensive plan, discussed in detail below, for building the capacity of South Sudan’s new NMRA, as well as the new NMRAs of Rwanda and Zanzibar and the soon-to-be new NMRA of Burundi.

In addition, the Roadmap calls for the initiative’s scope to widen to include new types of medical products, such as in vitro diagnostics (IVDs), vaccines, biologics and biosimilars, and medical devices. (Biologics are products made from living organisms or that contain components of living organisms, whereas biosimilars are officially approved versions of original "innovator" biological products, produced according to the same standards of pharmaceutical quality, safety, and efficacy; a biosimilar can be manufactured when the original product's patent expires.) For maximum efficiency, the program will concentrate on medical products that are not eligible for the WHO’s Prequalification Programme [9]. The WHO Prequalification Programme was primarily set up to evaluate and recommend products that meet WHO standards of quality and efficacy to UN procurement agencies and other global health product procurers (e.g., the Global Fund, UNICEF, GAVI). The WHO’s Prequalification Programme for IVDs, for example, prioritizes tests for HIV, hepatitis B and C, malaria, human papillomavirus, cholera, and syphilis [10]. Many emerging IVDs that could potentially be useful in the EAC, such as culture-independent assays to rapidly detect sepsis and analyze the antibiotic susceptibility of the organisms responsible [11] and rapid antibody tests for loiasis, a skin and eye disease caused by a parasitic worm [12], are not currently eligible for the Prequalification Programme—and access to IVDs such as these could be improved by the presence of a robust regional regulatory framework. By the end of 2020, the initiative aims to define guidelines for assessing novel IVDs and to include IVDs within the scope of its joint assessments; by 2022, it aims to have performed joint assessments of at least 10 IVD products.

The initiative will also expand into regulating vaccines that are not eligible for the WHO’s Prequalification Programme [13]. The shingles vaccine, for example, is not listed on the WHO’s priority list for its Prequalification Programme, but access to it could improve quality of life for many of the EAC’s older citizens. Thus, the ability to assess joint marketing applications for this and other vaccines is an important step in building the EAC’s regulatory capacity. In August 2019, the initiative finalized guidelines for assessing novel vaccines. By early 2020, the program will include vaccines within the scope of its joint assessments, and by 2022, it aims to have performed joint assessments of at least 10 vaccines.

Expanding into the assessment of biologics and biosimilars is also important for ensuring that EAC residents have access to the newest and most effective medicines. The WHO recently launched a new Prequalification Programme for biotherapeutics, but it is currently only accepting applications for two agents (and their biosimilars): rituximab, used principally to treat hematologic malignancies, and trastuzumab, used principally to treat breast cancer [14]. The EAC has the opportunity to improve access to other biologics and biosimilars, which are used to treat conditions as diverse as arthritis, psoriasis, multiple sclerosis, and diabetes, by beginning to assess these types of products. By 2022, the initiative aims to finalize and implement guidelines for assessing novel biologics and biosimilars. Because assessing applications for biologics and biosimilars requires special expertise, all applications for these products will be routed to joint assessments, in which two NMRAs can combine their expertise and skills with those of technical partners to produce a robust assessment that all the EAC’s NMRAs can use to make national registration decisions.

Finally, the initiative will expand into regulating medical devices. The WHO’s Prequalification Programme currently covers very few devices (e.g., immunization devices, male circumcision equipment) [15,16]. However, many other medical devices, such as pacemakers and infusion pumps, would be helpful to the people of the EAC, and the initiative can improve access to such devices through a joint assessment program. To this end, the initiative aims to adopt guidelines for assessing medical devices by 2022.

Tanzania’s NMRA will spearhead expansion into these new product areas. It currently leads the Medicines Evaluation and Registration Working Group, which will now encompass the registration of vaccines and biologics/biosimilars as well. Going forward, it will also lead one working group on IVD registration and one on medical device registration. In developing guidelines for IVDs and medical devices, these working groups will benefit from the progress made by the Pan African Harmonisation Working Party on Medical Devices and Diagnostics (PAHWP). The founding members of PAHWP, which is housed under AUDA-NEPAD, include the EAC’s Health Secretariat and Partner States, as well as Ethiopia, Nigeria, South Africa, and the London School of Hygiene & Tropical Medicine. With the help of partners such as the German Agency for International Cooperation, African Society for Laboratory Medicine, and WHO, PAHWP has been developing a common registration file to use for IVDs and medical devices, harmonizing rules for assigning a level of regulatory control that matches the risk these products pose to public health, and harmonizing guidelines for assessing these products and auditing the quality systems of product manufacturers [17]. Again, the initiative’s expansion in this area would encompass products not eligible for assessment by the WHO Prequalification Programme.

The last area in which the Roadmap calls for the initiative to expand is in the scope of its regulatory activities. The original vision was for the EAC MRH initiative to extend from pre-marketing activities, such as overseeing clinical trials, through product marketing application assessments and GMP inspections, to post-marketing safety and quality surveillance. By 2022, the initiative plans to realize this vision.

The need for effective, efficient clinical trial oversight in the EAC is pressing; in particular, the region needs a harmonized, transparent system with common requirements and standards. Without such a system, trial sponsors struggle to meet disparate requirements in different countries, making the region unattractive for trials. Trial sponsors often cite regulatory difficulties as a major reason for not undertaking trials across Africa. If one wants to undertake a trial, the regulatory guidelines are not readily available and differ from country to country in significant ways. Anyone seeking to carry out trials must spend valuable time understanding these different requirements and standards, and must submit different protocols in different countries for multicountry trials. When this barrier is added to other barriers, it makes Africa unattractive to trial sponsors. This is not a good state of affairs, as it means therapies get licensed without taking into account the unique genetic makeup of Africans or the environments and healthcare systems in which they live. These issues impact the appropriate dosing for a drug as well as its adverse event profile, yet important evidence on these topics emerges only after a product has been in use for several years in Africa, placing African patients at a disadvantage. As of June 2019, 332 clinical trials were registered in the EAC, 42 of them (13%) in multiple EAC Partner States [18]. The African Vaccine Regulatory Forum (AVAREF), which recently played a key role in expediting clinical trials of candidate Ebola vaccines and medical therapies [19], has already developed guidelines for the oversight of multiregional clinical trials in Africa [20]. These guidelines, along with the new Multi-Regional Clinical Trial guidelines released by the International Council for Harmonisation of Technical Requirements for Pharmaceuticals for Human Use [21], form a strong basis for the regional assessment of proposed clinical trials in Africa. The EAC plans to adapt these guidelines, using them to set minimum requirements for the oversight of any clinical trial that is conducted within its borders. To do so, it will rely on the expertise of a working group on clinical trials led by Burundi. By the end of 2020, the initiative plans to finish adapting the AVAREF guidelines, begin conducting joint evaluations of trials that span multiple EAC Partner States, develop guidelines for inspecting the contract research organizations (CROs) that conduct clinical trials, and begin conducting joint inspections of CROs.

The initiative will also expand its pharmacovigilance work, to better ensure access to safe and effective medicines. In March of 2019, the EAC Sectoral Council of Ministers approved the EAC Harmonized Compendium of Guidelines for Pharmacovigilance [22]. These harmonized guidelines were created by a working group on pharmacovigilance led by Kenya’s NMRA and include procedures and policy tools for reporting and sharing information on potential adverse reactions associated with registered medicines. In addition, Kenya, Tanzania, and Uganda are official members of the WHO Programme for International Drug Monitoring. They regularly send case reports of suspected adverse reactions to VigiBase, where the reports can be analyzed alongside those from other sources to determine whether countries should be alerted of potential safety signals. We expect that the addition of pharmacovigilance activities to the EAC MRH initiative will increase the number of EAC countries reporting adverse events to VigiBase, as well as the overall number of reports.

The initiative is also committed to increasing its laboratory field surveillance activities in order to help assure the quality of medicines on the market in EAC countries. Of note, Kenya, Tanzania, and Uganda are three of only five countries in sub-Saharan Africa with WHO-prequalified laboratories for medicine quality testing [23]. Moreover, all EAC partner states except for South Sudan have reported information to the WHO Global Surveillance and Monitoring System for Substandard and Falsified Medical Products, to identify and alert others about the presence of substandard and falsified products on the global market [24]. A program working group devoted to post-marketing quality surveillance, also led by Kenya, is currently developing a harmonized protocol for joint lab testing of field samples of medicines. Recently, with funding from Physikalisch-Technische Bundesanstalt, the EAC MRH initiative completed a study of the quality of antibiotics circulating in EAC Partner States, analyzing a little over 200 samples [25]; the findings resulted in the recall of one batch of drugs, and the knowledge gained will provide a foundation for further quality-surveillance activities in the EAC.

### Enhancing existing regulatory activities and building capacity

While beginning to assess new products and initiating new regulatory activities, the program will also strengthen and further optimize the activities it has performed from the start. Numerous improvements are planned for joint product assessments and GMP inspections. For example, in line with the Roadmap, in 2019 the initiative developed standard operating procedures (SOPs) for the joint processing of product variations and renewals, in response to a frequent request from medicine manufacturers active in the EAC. In addition to satisfying user demand, the ability to process product renewals more efficiently will help ensure that products on the EAC market retain their quality and are appropriately labeled. The initiative hopes that with these improvements and the enthusiasm and dedication of the program’s RTOs, by 2022 joint activities will become part of the everyday work of each NMRA, and that the number of joint assessments and GMP inspections will grow every year.

NMRA capacity building will also continue to be a major focus. The initiative will continue to design an IMS that connects all seven of the EAC’s NMRAs. This will entail setting up an IMS in South Sudan, as well as extending the regional IMS to handle payment, post-marketing surveillance, and clinical trial data. In terms of quality management, the initiative aims to have Rwanda, Burundi, and South Sudan achieve ISO 9001:2015 certification by 2022 so that all Partner States are on an even ISO level. Several Partner State NMRAs will also seek to receive Pharmaceutical Inspection Cooperation Scheme (PIC/S) or WHO certification for their GMP inspection programs by 2022. All NMRAs except for that of South Sudan will undergo formal WHO Global Benchmarking Tool assessments by 2022 as well, with the goal of each NMRA reaching a maturity level of 3 or higher. (According to the WHO’s Global Benchmarking Tool, Maturity Level 1 implies that there are some elements of a regulatory system existing in an agency; Maturity Level 2 means there is an evolving national regulatory system that partially performs essential regulatory functions; Maturity Level 3 indicates a stable, well-functioning, and integrated regulatory system; and Maturity Level 4 describes a regulatory system that is operating at an advanced level of performance and continuous improvement [26].) Tanzania’s NMRA recently became the first in Africa to attain this maturity level. Finally, the program will work with partners such as the WHO and well-resourced regulatory authorities outside of the EAC, such as the European Medicines Agency, to continue developing its staff’s expertise in regulatory affairs, and it will also develop a partnership with at least one university outside of the EAC that has a strong regulatory science curriculum.

### Creating the financial infrastructure to sustain the initiative

To date, the EAC MRH initiative has been financed primarily through donor funds (private foundations and bilateral and multilateral organizations). To ensure that the program can sustain its current work and the growth it has planned, it must transition to a system in which it becomes self-sustaining. The funding required is relatively modest. The Boston Consulting Group has estimated that it will cost approximately US$500,000 per year to maintain the initiative in its current state and roughly US$1.8 to US$2.6 million per year to set up and run the EAC Medicines Agency [5]. However, securing adequate funding is viewed by many of the initiative’s stakeholders as the most challenging part of its transition from a pilot program to a durable institution. Currently, the initiative plans to support itself with a mixture of application fees and contributions from Partner States’ NMRAs, although additional donor funding may be required to establish the EAC Medicines Agency.

Industry representatives have expressed eagerness to pay higher fees for product registration applications in order to fund a system that is robust, predictable, accountable, and transparent, with a single point of contact. This suggests that once an optimized process for regional product assessments and GMP inspections is fully operational, the initiative could support itself by levying regional coordination fees for joint activities (Fig 2). One analysis estimated that 60 regional applications a year could potentially support an RTO-led coordination desk for regional assessments and inspections, and 145 regional applications a year could support an EAC Medicines Agency [5]. Thus, it seems reasonable that as the frequency of joint activities grows, the initiative can eventually support itself without donor funding, just as some of the EAC’s more mature NMRAs are already supporting themselves almost solely through the fees they take in [27]. By the end of 2021, the initiative aims to have implemented this new revenue model financed primarily by regional activities. Industry paying fees for service to regulators is a global standard used by a majority of regulatory agencies, including the United States Food and Drug Administration (FDA), the European Medicines Agency, and all regulatory agencies in East Africa. These fees are generally linked to a client charter, which sets expectations in terms of the services to be provided and key performance indicators. The fees for marketing authorization, for example, cover the service of assessing an application and providing a decision (accept or reject); the fee has nothing to do with the outcome of the review.

## Conclusion

The EAC MRH initiative’s Roadmap describes many paths to a stronger, more efficient, and more accountable region-wide regulatory system. Although some changes, such as establishing an EAC Medicines Agency, will take years, numerous critical changes will take place in the next year or two. These key improvements will include the following:

- More robust and predictable joint product assessments and GMP inspections, which will result in more efficient national registrations
- The improved capacity of all NMRAs to engage in critical regulatory activities
- Increased revenue driven by an increase in joint activities

Both the short- and long-term improvements planned during the next phase of the initiative will have an immediate impact on the ability of EAC residents to access quality, safe medicines. These improvements will also help pave the way for establishing a continent-wide initiative to harmonize technical regulatory standards for medicines and to optimize regulatory processes, in the form of the African Medicines Agency (AMA), whose treaty was endorsed by the heads of government of the African Union in 2019 and is expected to be ratified in the next 2 to 3 years. Although we recognize that the EAC MRH initiative’s plans for the future may seem ambitious, we believe they are feasible, given the program’s accomplishments during its pilot phase. We look forward to reporting the program’s progress in future publications, and we hope to hear from regulatory experts in other regions of the world who are interested in sharing ideas about how to improve regional approaches to delivering more robust, efficient, predictable, accountable, and sustainable regulatory activities.

## Abbreviations

AMA: African Medicines Agency
AUDA-NEPAD: African Union Development Agency–New Partnership for Africa’s Development
AVAREF: African Vaccine Regulatory Forum
CRO: contract research organization
EAC: East African Community
FDA: United States Food and Drug Administration
GMP: good manufacturing practice
IMS: information management system
IVD: in vitro diagnostic
MRH: Medicines Regulatory Harmonization
NMRA: national medicines regulatory authority
PAHWP: Pan African Harmonisation Working Party on Medical Devices and Diagnostics
PIC/S: Pharmaceutical Inspection Cooperation Scheme
RTO: regional technical officer
SOP: standard operating procedure
Swissmedic: Swiss Agency for Therapeutic Products
WHO: World Health Organization
